# The Chemokine CX3CL1 (Fractalkine) and its Receptor CX3CR1: Occurrence and Potential Role in Osteoarthritis

**DOI:** 10.1007/s00005-014-0275-0

**Published:** 2014-02-21

**Authors:** Piotr Wojdasiewicz, Łukasz A. Poniatowski, Andrzej Kotela, Jarosław Deszczyński, Ireneusz Kotela, Dariusz Szukiewicz

**Affiliations:** 1 Department of General and Experimental Pathology, Second Faculty of Medicine, Medical University of Warsaw, Pawinskiego 3c, 02-106 Warsaw, Poland; 2 Department of Orthopaedics and Rehabilitation, Second Faculty of Medicine, Medical University of Warsaw, Warsaw, Poland; 3 Department of Orthopaedics and Traumatology, Central Clinical Hospital of the Ministry of the Interior, Warsaw, Poland

**Keywords:** Chemokine CX3CL1, Fractalkine, Fractalkine receptor, CX3CR1, Osteoarthritis

## Abstract

Chemokines are molecules able to induce chemotaxis of monocytes, neutrophils, eosinophils, lymphocytes and fibroblasts. The complex chemokine acts in many physiological and pathological phenomena, including those occurring in the articular cartilage. To date, chemokine CX3CL1 (fractalkine) is the only member of the CX3C class of chemokines with well-documented roles in endothelial cells. CX3CL1 is a unique chemokine that combines properties of chemoattractant and adhesion molecule. The main roles of CX3CL1 include promotion of leukocyte binding and adhesion as well as activation of the target cells. The soluble chemokine domain of CX3CL1 is chemotactic for T cells and monocytes. CX3CL1 acts via its receptor, CX3CR1, which belongs to a family of G protein-coupled receptors. Stimulation of CX3CR1 activates both CX3CL1-dependent and integrin-dependent migrations of cells with synergistically augmented adhesion. Genetic polymorphisms of CX3CR1 may significantly modify the biological roles of CX3CL1, especially in pathologic conditions. Osteoarthritis (OA) is the most common joint disease, affecting approximately 7–8 % of the general population. Development of OA is largely driven by low-grade local background inflammation involving chemokines. The importance of CX3CL1/CX3CR1 signalling in the pathophysiology of OA is still under investigation. This paper, based on a review of the literature, updates and summarises the current knowledge about CX3CL1/CX3CR1 in OA and indicates possible interactions with a potential for therapeutic targeting.

## Introduction

Osteoarthritis (OA) is the most common chronic joint disease (Bijlsma et al. 2011). OA occupies a special place among musculoskeletal conditions. OA is characterised by a number of factors, including epidemiological parameters, a multifactorial aetiology, a complex pathogenesis and troublesome clinical manifestations as well as the still-unsolved problem of providing optimum treatment (Arden and Leyland 2013; Wieland et al. 2005). A characteristic feature of OA is a sequence of pathological changes that is drawn out in time and involves all joint-forming tissues. A unique combination of pathophysiological and biochemical abnormalities gradually induces proliferation, degradation and deformation processes in the affected joint. These changes present, among others, as sclerosis of the subchondral layer of bone, loss of cartilage tissue and remodelling of joint surfaces, and may induce osteophyte formation (Loeser et al. 2012). All these pathologies gradually impair joint mobility, aggravate instability and cause increasing pain, thus producing significant deterioration of the patient’s quality of life.

In recent years, independent authors looking to embrace a possibly comprehensive explanation of the pathogenesis of OA in their papers have been devoting increasingly more attention to the importance of an inflammatory component (Haseeb and Haqqi 2013; Sokolove and Lepus 2013). Advances in the basic and clinical sciences over the last 20 years have substantiated the belief that OA is not merely a degenerative disease associated with loss of articular cartilage, but that a wider approach is needed—including a systemic angle—in view of a significant contribution of the immune response (Haseeb and Haqqi 2013; Scanzello and Goldring 2012). The inflammatory component is an integral element of OA at both its early and advanced stages (Benito et al. 2005; Haseeb and Haqqi 2013). Current research most often focuses on mechanisms involving inflammatory mediators (such as cytokines, including chemokines) secreted by cells within the joint and those infiltrating joint structures from blood vessels (Chevalier et al. 2013).

Chemokines are a family of low molecular weight proteins involved in chemotactic (as their name suggests) control of the migration of leukocytes and other cells involved in inflammation. Among more than 50 cytokines with chemotactic properties described to date, the chemokine CX3CL1 (fractalkine) merits special attention. While sharing some properties typically seen in other chemokines, it has a unique molecular structure and may act as an adhesion molecule. To date, CX3CL1 has been investigated with its occurrence and potential involvement in the pathophysiology of OA regarded only in few studies. Accordingly, it is advisable to present, in a review of the literature, current knowledge about the contribution of CX3CL1 and its receptor, CX3CR1, to OA.

Importantly, OA is not the only chronic joint disease that CX3CL1 plays a role in. There is ample literature on the occurrence and significance of this chemokine in other rheumatic diseases, particularly in rheumatoid arthritis (RA) (Blaschke et al. 2003; Jones et al. 2012; Nanki et al. 2002; Umehara et al. 2006; Volin et al. 2001). In RA patients, fractalkine has been detected in the synovial fluid, in peripheral blood and in inflamed synovial membranes, often at concentrations several times higher than those seen in other arthropathies, including OA (Endres et al. 2010; Nanki et al. 2002; Ruth et al. 2001; Yano et al. 2007). At the same time, the occurrence and potential role of CX3CL1 in the development of OA has not been summarised in a review paper it certainly “deserves”; to our knowledge, the present paper is the first comprehensive review specifically devoted to this issue. The review is preceded by a general update on chemokines and on the position of CX3CL1 within this family of compounds.

## Chemokines: Cytokines with Chemotactic Properties

Cytokines are a class of glycoprotein compounds acting as cellular hormones. They are released by activated cells to exert local and/or systemic effects on various kinds of human cells and tissues. The increasingly more numerous family of cytokines includes the subfamily of chemokines, distinguished in view of their unique structural and chemoattractant properties (Colobran et al. 2007). The name chemokines was accepted at a congress in Baden in 1992 by consensus of international research groups (Taub and Oppenheim 1993; Richmond 2011). The establishment of a uniform division of chemokines has served to embrace the rapidly growing number of newly discovered compounds of this class in the 1990’s and the gradual broadening of knowledge about their mechanisms of action (Taub and Oppenheim 1993; Richmond 2011; Zlotnik and Yoshie 2012). Chemokines are low molecular weight proteins that form a “chemokine network” (Colobran et al. 2007). The division of chemokines into subgroups can be based on functional or structural criteria (Mortier et al. 2012; Zlotnik and Yoshie 2012). The functional division classifies compounds based on whether they are produced constitutively under homoeostatic conditions or formed as part of a stress reaction during which chemokines modulate the immune response (Mortier et al. 2012; Zlotnik and Yoshie 2012). In accordance with this criterion, chemokines can be divided into homoeostatic, proinflammatory, platelet- and plasma-associated and, finally, those that can exert all these effects at the same time. According to the latest classification of 2012, chemokines are divided structurally into four basic subfamilies: (X)C, CC, CXC and CX3C (Zlotnik and Yoshie 2012). The main underlying criterion is the mutual arrangement of amino acids, cysteine residues and disulphide bridges within the molecule. Interactions within the chemokine network occur as a result of the affinity of one chemokine to various types of receptors and the activation of a specific receptor by several chemokine ligands. This phenomenon may be viewed as a way to safeguard chemokine-mediated processes in cases of dysfunction or lack of a given ligand or receptor. It is owed to multiple duplication of the genes coding for chemokine proteins and receptors and to evolutionary mutations (Nomiyama et al. 2010). Ligand receptor interactions activate a number of intracytoplasmic pathways leading to functional and morphological changes in the cell (Mellado et al. 2002; Muñoz et al. 2012). Thus, a cell’s response to chemokines is influenced by the cellular phenotype and the multiplicity of combinations of the receptor-ligand complex.

The main role of chemokines in the body is modulation of the immune response and chemotactic action on leukocytes by way of creating a concentration gradient (Johnston and Butcher 2002; Mortier et al. 2012; Richmond 2011; Zlotnik and Yoshie 2012). Research on the properties and function of chemokines in the human organism over the last 20 years has brought a number of discoveries that, among others, identify chemokines as key players in the pathophysiology of inflammation and many other processes, such as organogenesis (Joseph et al. 2010), angiogenesis (Owen and Mohamadzadeh 2013), haematopoiesis (Broxmeyer 2008), immunity to infection (Wolf and Moser 2012), atherosclerosis (Braunersreuther et al. 2007), carcinogenesis (Mantovani et al. 2010) and autoimmune response (Proost et al. 2006). The variability of genetic sequences, discussed above, suggests the presence of substantial polymorphism among chemokine receptors, which accounts for intra- and interindividual differences in overt effects of chemokines (Colobran et al. 2007; Nomiyama et al. 2010). Another conclusion related to this genetic variability is that the risk of development of particular diseases will vary among members of a population and the immune response in the course of these diseases may differ considerably between individuals or even have unique features (Colobran et al. 2007; Nomiyama et al. 2010). Known properties of chemokines are still the subject of detailed studies, offering hope for the development of modern targeted therapies.

## CX3CL1

**Fig. 1:**
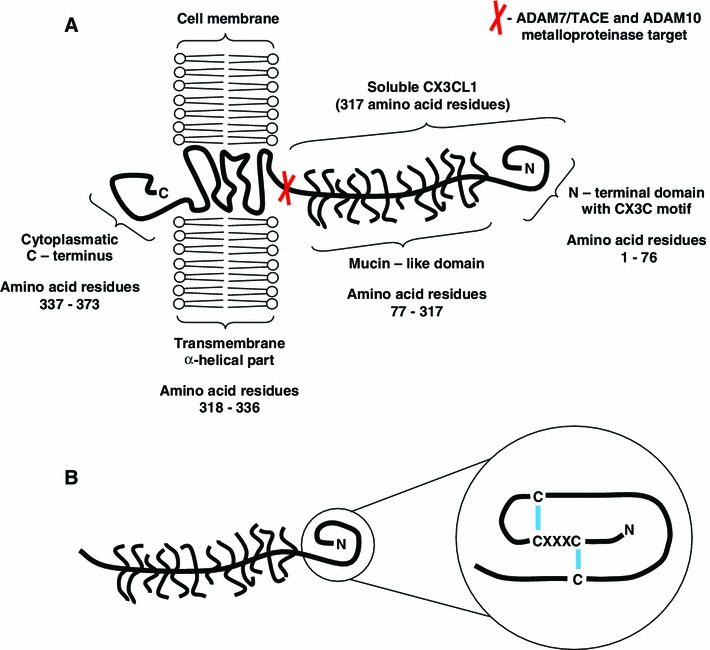
The molecular structure of the membrane-bound form of CX3CL1 (fractalkine) showing specific regions of the molecule and the site of the cleaving action of the metalloproteinases ADAM7/TACE and ADAM10 (**a**). The unbound form of fractalkine, produced by metalloproteinase cleaving. The domain containing the CX3C motif is shown in greater detail and the disulphide bonds are marked in *blue* (**b**)

Among the approximately 60 compounds in the family of chemokines, special attention is due to be paid to the molecule known as CX3CL1 and its receptor CX3CR1 (previously called V28). The name fractalkine was first used to describe a newly discovered chemokine by Bazan et al. (1997). A few weeks later, the discovery was confirmed by Pan et al. (1997), who used the name neurotactin. Under the current nomenclature, with regard to structure, CX3CL1 is the only representative of the CX3C (δ) subfamily (Zlotnik and Yoshie 2012). In the human genome, the CX3CL1 gene occupies the locus 16q13 (Nomiyama et al. 1998). The ultrastructure of CX3CL1 is unique in including a motif composed of three amino acid residues between two cysteine residues forming the disulphide bridges that stabilise the tertiary structure of the molecule (Bazan et al. 1997; Kim et al. 2011). Two CX3CL1 isoforms occur: a soluble one (cytosol-associated) and a form tethered to cell membranes (membrane-anchored CX3CL1; Fig. 1) (Bazan et al. 1997; Kim et al. 2011). The existence of two forms of CX3CL1 in the body accounts for its special role in the chemokine network that has not been paralleled by any other chemokine to date.

Soluble CX3CL1 is a chemotactic factor for, among others, NK cells (Hamann et al. 2011), T cells (Mionnet et al. 2010), monocytes (Ancuta et al. 2003) and mast cells (Papadopoulos et al. 2000). In addition, soluble CX3CL1 contribution has been proven in angiogenesis and endothelial cell chemotaxis (Volin et al. 2010). Unbound CX3CL1 does not chemoattract neutrophile populations, while its membrane-bound form, present predominantly in vascular endothelial cells, mediates neutrophile binding and adhesion. Not surprisingly, CX3CL1 expression is increased in richly vascularised and well-innervated organs and at sites of increased leukocyte concentration, although it is highly specific for particular cell types (Kim et al. 2011). Local CX3CL1 synthesis and expression is regulated by a number of factors, such as proinflammatory cytokines (interleukin (IL)-1β, interferon (IFN)-γ, tumour necrosis factor (TNF)-α), the presence of lipopolysaccharide, tissue oxygen pressure and auto- and paracrine autoregulation (Imaizumi et al. 2004; Zhu et al. 2011; Zujovic et al. 2000). All these factors activate a network of intracellular messengers and transcription factors, leading to increased or reduced CX3CL1 production (Cambien et al. 2001).

## The CX3CR1 Receptor

The biological effects of CX3CL1 are the result of its interaction with the CX3CR1 (formerly V28) receptor (Imai et al. 1997; Kim et al. 2011; Mizoue et al. 1999). Structurally, the CX3CR1 receptor belongs to the class of metabotropic receptors, also known as G protein-coupled receptors, or seven-transmembrane proteins (Kim et al. 2011).

**Fig. 2:**
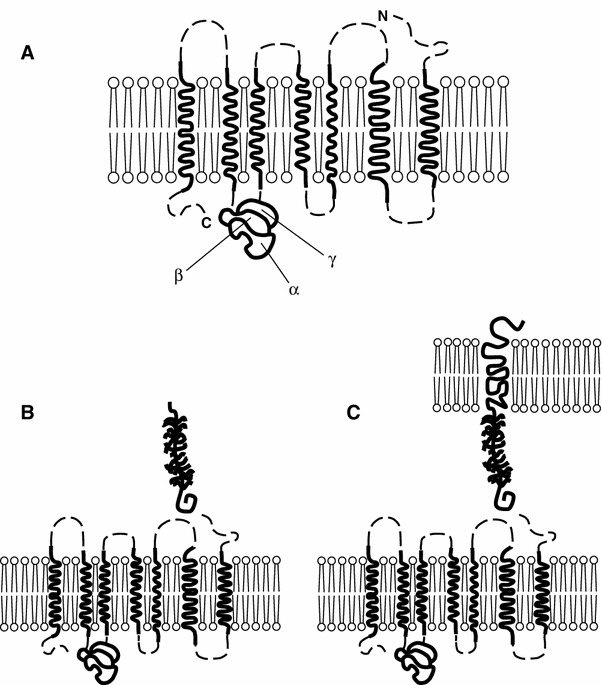
Structure of the CX3CR1 receptor showing the 7 transmembrane α-helixes forming the receptor protein and the individual subunits of the heterotrimeric protein G the receptor is coupled with (**a**). The free form of CX3CL1 can interact with the CX3CR1 receptor (**b**). The receptor interacting with the membrane-bound form (**c**)

**Fig. 3:**
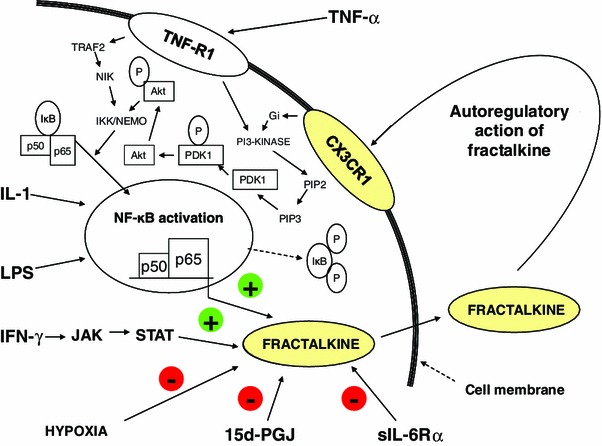
Selected signal pathways producing a local increase (+) in CX3CL1 levels via activation of nuclear factor (NF)-κB) or producing inhibition (–) of CX3CL1 production. Shown in detail is the signal pathway associated with TNF-R1 (type 1 receptor for TNF-α) and CX3CR1 (CX3CL1 receptor). Of importance is the autoregulatory nature of the interaction between CX3CL1 and CX3CR1. Published by courtesy of Dr. Mittal (2012). *LPS* lipopolysaccharide (endotoxin), *JAK* janus kinase, *STAT* signal transducers and activators of transcriptions, *15d-PGJ* 15-deoxy-Δ12,14-prostaglandin J, *sIL-6Rα* soluble form of the subunit α of the IL-6 receptor, *p50, p60* subunits of proteins forming NF-κB, *IκB* (inhibitor of κB)—an endogenous complex of proteins inhibiting the activation of NF-κB, *P* symbol of phosphorylation, *PDK1* pyruvate dehydrogenase kinase 1, *Gi* G inhibitor protein, *PI3* phosphatidylinositol-3-kinase, *PIP2* phosphatidylinositol 4,5-bisphosphate, *PIP3* phosphatidylinositol 3,4,5-triphosphate, *TRAF2* TNF receptor-associated factor 2, *NIK* NF-κB-inducing kinase, *Akt* serine-threonine protein kinase, *IKK/NEMO* NF-κB inhibitor kinase (Iκβ kinase)/NF-κB kinase inhibitor (NF-κB essential modulator)

The polypeptide chain is made up of seven α-helical structures extending across the thickness of the cell membrane. Accordingly, an extracellular, transmembrane and intracellular part of the receptor can be distinguished (Fig. 2). External loops of the polypeptide chain form the binding site for ligands CX3CL1 (Mizoue et al. 1999) and CCL26 (Nakayama et al. 2010). Intracellular loops in the cytoplasm and the C-terminal end of the chain form the site that the heterotrimeric protein Gαi attaches to. The CX3CR1, similar to other chemokine receptors, displays polymorphism, which may account for its varying affinity for CX3CL1 and offer possibilities in choosing targeted therapeutic interventions (Niessner et al. 2005). A fundamental discovery in research on the CX3CR1 receptor was the finding that CX3CL1 has autoregulatory function and that it interacts with the TNF type 1 receptor to activate the nuclear transcription factor κB (Fig. 3). Importantly, the existence of one receptor for CX3CL1 makes it much easier to interpret the observed biological effects of this chemokine.

## Occurrence of CX3CL1 and its Receptor, CX3CR1, in Affected Tissues in the Course of OA

Despite a proven role of CX3CL1 within the so-called chemokine network, existing studies of its significance in inflammatory joint disease have neglected the most common degenerative joint disorder. Available data from the literature, while scant, confirm that CX3CL1 and its receptor are involved in OA. An analysis of the information presented below may help to better understand the complex and multifactorial pathomechanisms of OA and, in the future, may aid in efforts to develop new treatment methods.

Ruth et al. (2001) were the first to detect soluble CX3CL1 in synovial fluid of arthropathic patients. Their ELISA-based study involved patients with OA and other rheumatic diseases (RA, juvenile RA, psoriatic arthritis, polyarthritis, spondyloarthropathy, inflammatory myopathy and gout). The findings revealed soluble CX3CL1 levels of 1.4 ± 0.4 ng/ml (*n* = 13) in OA patients.

In another study, Nanki et al. (2002) investigated CX3CL1 levels and CX3CR1 receptor expression in surgically harvested synovial membranes. This study involved four patients with OA and seven patients with RA. Following immunohistochemical staining of synovial membrane specimens, the authors confirmed CX3CR1 expression in both OA and RA. CX3CL1 was not detected in synovial membranes of either OA patients or RA patients. Blaschke et al. (2003) confirmed the earlier findings of Nanki et al. (2002) regarding the expression of CX3CL1 and CX3CR1 in tissues obtained from OA patients.

The occurrence of soluble CX3CL1 in peripheral blood and synovial fluid was investigated by Yano et al. (2007). Samples from 12 healthy individuals (controls), 16 patients diagnosed with OA and 22 patients with RA were subjected to an immunoenzymatic test. Mean serum CX3CL1 levels were 0.56 ± 0.56 ng/ml in the control group vs. 8.00 ± 4.46 ng/ml in OA patients, representing a statistically significant elevation in the latter group. Mean CX3CL1 concentration in synovial fluid in OA patients was 3.17 ± 2.48 ng/ml. Samples of synovial fluid were not obtained in the control group and CX3CL1 levels in synovial fluid in the OA group were only compared to respective data in RA patients (34.32 ± 15.36 ng/ml).

Leonov et al. (2011) used the ELISA test to study the correlation between OA and circulating CX3CL1 levels in venous blood. The study enroled 923 patients, of whom 187 constituted the core group of 130 patients with intervertebral disc disease (ICD-10: M51.3) and 57 patients with OA (of the knee, shoulder, hand and other joints). The remaining 736 patients were not diagnosed with any arthropathy on examination and they constituted a control group. The study showed that circulating CX3CL1 levels in the blood were significantly elevated in the core group as compared to the control group.

The correlation between radiographic progression of osteoarthritic lesions according to the Kellgren-Lawrence grading scale (KLGS) (Kellgren and Lawrence 1957) and CX3CL1 levels in venous blood and synovial fluid was analysed by Zou et al. (2013) in a study of 223 patients with osteoarthritis meeting the criteria of the American College of Rheumatology (Altman et al. 1986). A control group consisted of 165 healthy individuals without a history of joint disease. First, CX3CL1 levels in peripheral blood were compared between the OA patients and controls. Mean fractalkine concentration in the OA group was 226.25 pg/ml, as compared to 127.42 pg/ml in the control group. Mean CX3CL1 concentration in synovial fluid in the OA group was 81.87 pg/ml. However, it should be mentioned that level of CX3CL1 in the OA synovial fluid was much lower than in OA and healthy serum. This may indicate that the CX3CL1 production is situated outside the area affected by OA joints and should be a subject of further studies. In the same study, CX3CL1 levels in the serum and synovial fluid of OA patients were compared with regard to radiographic severity of OA lesions according to the KLGS. There was a correlation between increasing serum and synovial fluid CX3CL1 levels and increasing severity of osteoarthritic lesions on radiographs. Accordingly, the authors note that CX3CL1 may potentially serve as a marker of OA progression.

Beekhuizen et al. (2013) analysed the presence of inflammatory mediators in knee synovial fluid. Inflammatory mediators were detected by immunoenzymatic assays (ELISA). The study enroled 18 patients with known OA of the knee joints diagnosed in accordance with the American College of Rheumatology criteria (Altman et al. 1986). A control group consisted of 16 deceased individuals in whom OA was ruled out and samples of synovial fluid were obtained within 24 h of death. CX3CL1 was not detected in any sample of synovial fluid in the control group, and in the OA group CX3CL1 concentrations were in the range of 0 ± 19 pg/ml (median ± interquartile range). A similar study had been conducted earlier by Endres et al. (2010). Both studies confirmed the presence of CX3CL1 in the synovial fluid of OA patients and absence of this chemokine in synovial fluid collected from non-OA controls.

## Potential Role of CX3CL1 and its Receptor, CX3CR1, in the Pathophysiology of OA

Research by Klosowska et al. (2009) aimed to gain more knowledge about the role of the CX3CL1/CX3CR1 signalling pathway in the complex of cells involved in the development of OA. The resulting paper (Klosowska et al. 2009) described the effect of CX3CL1 on synovium-associated fibroblasts in OA patients. A number of in vitro experiments revealed that CX3CL1 had a chemotactic effect on fibroblasts resulting in their migration along the concentration gradient of CX3CL1. Investigations of the structure of F-actin in fibroblasts temporarily exposed to CX3CL1 revealed structural reorganisation of the cytoskeleton of these cells enabling them to move towards the stimulus. An additional experiment testing the influence of CX3CL1 on cytokine production by fibroblasts revealed no significant effect on the quantity and/or profile of cytokines produced. The observed response of fibroblasts to the presence of CX3CL1 leads to the conclusion that fibroblasts express functional CX3CR1. The possibility of CX3CL1-mediated activation of mitogen-activated protein kinases was also considered. Activation of these intracellular messengers appears to play a significant role in the production by fibroblasts of particular proteins involved in the pathophysiology of OA and also to initiate the ability to avoid apoptosis (antiapoptotic effect) by activating the Akt kinase.

Chondrocytes forming joint cartilage are one of the most important cell groups directly involved in the development of OA. Sandell et al. (2008) conducted several studies on the effect of IL-1β on chondrocytes harvested from OA patients. IL-1β is a cytokine with known proinflammatory properties detected in OA patients (Scanzello and Goldring 2012). Among other findings, the study showed a mutual correlation between the action of IL-1β and increased expression of mRNA for the chemokine CX3CL1. RT-PCR testing revealed that even the lowest concentrations of IL-1β of the order of 0.01 ng/ml stimulated the chondrocytes to produce genes responsible for the production of CX3CL1, and increasing IL-1β levels (at 0.1 and 1 ng/ml) correlated with increases in the quantity of CX3CL1-specific mRNA. Interestingly, the increase in CX3CL1 mRNA expression in joint cartilage cells correlated with the time since administration of IL-1β, with increased levels of the CX3CL1 transcript seen circulating in the cytoplasm of chondrocytes as early as at 1 h following the administration of 0.1 ng/ml IL-1β. Peak expression was recorded at 4 h, following which a gradual downward trend was noted during successive measurements at 8, 12 and 24 h. These results may correspond with those of a third experiment, comparing the effect of IL-1β on normal chondrocytes (control group) and chondrocytes harvested from OA patients (experimental group). The latter, being constantly exposed to IL-1β in vivo, displayed less marked expression of CX3CL1 mRNA during re-exposure to IL-1β in vitro as compared to control chondrocytes, which had not been previously exposed to IL-1β. This suggests the existence of a down-regulation phenomenon in chondrocytes harvested from the OA patients, which, in turn, may indicate that CX3CL1 plays a more important role in stimulating the inflammatory process in the early stages of OA than at later stages.

Osteoblasts are a type of cells responsible for the formation of osseous structures and continuous biochemical bone turnover. Isozaki et al. (2008) studied osteoblasts harvested from OA patients and exposed simultaneously to TNF-α and IFN-γ, showing that the stimulation increased both the production of CX3CL1 mRNA and the synthesis of free CX3CL1. A comparison with a control group of similarly stimulated normal cells demonstrated a much more marked rise in the production of CX3CL1 mRNA and CX3CR1 by osteoblasts harvested from OA patients. These results may indicate that osteoblasts in OA patients, which are exposed to the synergistic effects of TNF-α and IFN-γ in the course of the disease, represent a major cellular source of CX3CL1 produced in affected joint structures.

## Conclusions

Osteoarthritis is an important problem in today’s ageing society. Its presentation includes both its multifactorial aetiology and a complex course as well as the ever-present problem of providing optimum treatment. All these factors make OA a continuing challenge to several medical specialities. On the basis of available international literature, we have discussed the potential role of CX3CL1 and the CX3CR1 receptor in the pathophysiology of OA. As stated in the introduction, the presence of CX3CL1 in synovial fluid, peripheral blood and inflamed tissues is not a finding confined to OA patients. Patients with other inflammatory joint conditions, such as RA, often demonstrate significantly higher CX3CL1 levels than patients with OA. The data presented in this paper, however, clearly imply that the presence and role of fractalkine in the development of OA should not be ignored by researchers. Independent studies from various centres confirm that elevated levels of CX3CL1 are present in synovial fluid of OA patients as compared to samples obtained from healthy controls. Of note is also the conspicuous correlation between CX3CL1 levels in synovial fluid and in peripheral blood. This correlation also makes it possible to link these parameters to progression of radiographic degenerative lesions. This paves the way for employing CX3CL1 as a helpful marker in determining the severity of OA or monitoring treatment outcomes. The presence of CX3CR1 has been described in the synovial membranes of OA patients. More detailed investigations shed light on potential phenomena on cellular and subcellular level in cells directly involved in the pathophysiology of OA. The few studies available so far are not sufficient to fully elucidate the role of CX3CL1 and CX3CR1 in the pathomechanism of OA. As stated in the introduction, assessment of the occurrence and role of CX3CL1 and its receptor requires further, more detailed studies that hold promise for tangible future benefits stemming from more precise identification and understanding of the nature of OA at cell level and, consequently, the development of new treatment methods.
